# Exploring relationship satisfaction in mothers of children with disabilities: the predictive role of interparental conflicts and moderating role of dyadic coping

**DOI:** 10.3389/fpsyt.2023.1307827

**Published:** 2024-01-08

**Authors:** Marijana Vrankić Pavon, Ana Wagner Jakab, Ajana Löw

**Affiliations:** ^1^Department of Inclusive Education and Rehabilitation, Faculty of Education and Rehabilitation Sciences, Zagreb, Croatia; ^2^Faculty of Education and Rehabilitation Sciences, Zagreb, Croatia

**Keywords:** parenting stress, interparental conflicts, dyadic coping, relationship satisfaction, developmental disabilities

## Abstract

**Background:**

Parenting responsibilities, while a source of happiness, often lead to considerable stress for parents of children with disabilities. While most parents try to cope with these challenges together, some level of interparental conflict is almost inevitable. Frequently assuming primary responsibility for children with disabilities, mothers can be substantially impacted by interparental conflict with their partners and the resulting stress. However, implementing dyadic coping strategies in managing stressful situations serves as a buffer against adverse effects, while also enhancing relationship satisfaction.

**Objective:**

This study aimed to explore the relationship between interparental conflicts, dyadic coping, and relationship satisfaction among 232 mothers of children with disabilities in Croatia who were in an intimate relationship and shared a household with their partners, regardless of marital status.

**Methods:**

We analysed a non-probabilistic sample of women in Croatia who reported being in an intimate relationship, sharing a household with a partner (whether married or simply cohabitating) and being a mother to a child with a diagnosed developmental disability. Participants completed questionnaires online in March of 2021 (sociodemographic variables, the Parent Problem Checklist, the Dyadic Coping Inventory and the Marriage Quality Index).

**Results:**

Results of hierarchical multiple regression show that mothers’ perceptions of how they help their partner cope with stress do not predict how satisfied they are in a relationship; instead, relationship satisfaction depends on their perceptions of how their partner helps them and how they cope with stress as a team. Furthermore, moderation analysis revealed the effect of interparental conflicts were entirely mitigated by high supportive and low negative dyadic coping of the partner. When mothers felt adequately supported by their partner, the negative impact of stress caused by interparental conflicts did not spill over into their relationship.

**Conclusion:**

Our results suggest that by recognizing the importance of psychological support and couples therapy in strengthening dyadic coping, government and non-profit initiatives can effectively empower parents of children with disabilities, fostering healthier and more resilient family dynamics that promotes the well-being of parents and their children.

## 1 Introduction

Even though children are a source of happiness for their parents, parenting responsibilities are among the greatest sources of stress for couples (1). The stresses associated with parenting can be analysed through the prism of family systems theory (2), which conceptualizes the family as a system of intimate relationships: between parents, between children, and between parents and children. Misalignment and tension in these intimate relationships increase parenting burden and reduce parenting effectiveness (3), while also harming the intimate relationships between parents (4, 5), reducing the frequency or quality of their interactions and behaviors to care for each other, accept each other, maintain the sexual dimension of their relationship, pursue joint activities and share responsibilities (6). Parenting stresses can also combine with external factors, such as financial problems or unemployment, to reduce intimate relationship satisfaction (7).

Parental stresses are often even stronger among parents raising children with disabilities (8–10), such as autism spectrum disorder or attention deficit hyperactivity disorder (11, 12) which is not unexpected since these children, in addition to core symptoms of disabilities, sometimes also have behavioral problems (13). Several studies indicate that child behavior difficulties predict greater parental stress, e.g., increasing risk of emotional and psychological distress (14, 15), which can reduce parenting effectiveness (16). Co-parenting can prove particularly challenging for mothers because, despite a tendency since the 1950s toward equal role-sharing between fathers and mothers (17), mothers of children with disabilities often perform the lion’s share of childrearing (18), which leads to reports of lower life satisfaction compared to fathers of the same children (19). Furthermore, interparental conflict is reciprocally related to child adjustment (e.g., internalizing and externalizing problems) (20), so in families with children facing disabilities, frequent interparental conflict may coincide with more challenging behaviors in the children, thereby intensifying stress for the parents.

Parental stress can lead to internal stress between parents, which increases interparental conflicts and decreases marital satisfaction (21). This process of external stress affecting the couple’s relationship is known as the spillover effect (22), which may have an even greater impact on parents of children with disabilities (21). One study (23) compared families of children with disabilities and families of typically developing children in terms of emotional regulation, parental stress and family functioning and they found greater parental stress, more interparental conflicts and lower relationship satisfaction in parents of children with different types of disabilities (e.g., intellectual disability, autism spectrum disorder, ADHD, Down syndrome, learning disabilities).

For such parents, a satisfactory intimate relationship can be critical for buffering the challenges of caring for children with disabilities (24). Co-parenting can preserve the relationship between parents and render it more resilient to stresses by strengthening dyadic coping between them, as described by the systematic transactional model (25). Dyadic coping (DC) has been associated with better relationship quality (26), higher relationship satisfaction (27), and greater relationship stability (28). It can buffer the negative effects of chronic stress (29), including stresses related to children (30). Stronger dyadic coping can decrease partnership conflict (31) and improve relationship satisfaction (32–34) and martial adjustment (35, 36).

Furthermore, dyadic coping is of great importance when it comes to coping with chronic stress (37). Parents of children with disabilities face stress and parenting challenges throughout their child’s life. It may be the most important process in adapting to life in chronic stress context. For example, parents of children with autism spectrum disorder face major parenting challenges due to the complex nature of autism. One study (38) researched parents of children with different types of disabilities (e.g., motor, intellectual, sensory and multiple disabilities) found that in addition to DC, the partner’s responses to relationship-focused coping are decisive. In other words, relationship-focused coping in the context of a perceived negative response from the spouse was associated with greater psychological distress, whereas in contrast, relationship-focused coping and distress in the context of a positive perception of the spouse’s responses to coping were not associated.

Parents raising children with disabilities are particularly vulnerable to the stresses and strains of parenting, and so is their marital relationship; yet we are unaware of studies exploring interparental conflicts, dyadic coping, and relationship satisfaction among mothers of children with developmental disabilities. We speculated that mothers of children with disabilities would experience parenting conflicts with their partners and that their relationship satisfaction would depend on conflict frequency, intensity, and dyadic coping quality. Older age and longer relationship duration might reduce relationship satisfaction; however, conflict frequency, intensity, and dyadic coping might be stronger predictors of satisfaction than sociodemographic variables. We aimed to investigate the following research hypotheses:

*H1*: Mothers’ age and duration of intimate relationship, as well as interparental conflicts and dyadic coping will predict relationship satisfaction of mothers of children with disabilities.

*H1.1*: Mothers’ age, duration of intimate relationship and interparental conflicts will negatively predict relationship satisfaction, with interparental conflicts being the strongest predictor.

*H1.2*: Subscales of dyadic coping (supportive and delegated coping of oneself and of the partner) will positively predict relationship satisfaction.

*H1.3*: Negative dyadic coping of oneself and of the partner will negatively predict relationship satisfaction.

*H2*: Dyadic coping will moderate the association between interpersonal conflicts and relationship satisfaction.

*H2.1*: Supportive dyadic coping will mitigate the association between interparental conflicts and relationship satisfaction.

*H2.2*: Negative dyadic coping will intensify the association between interparental conflicts and relationship satisfaction.

## 2 Method

### 2.1 Participants

The study analysed a non-probabilistic sample of women in Croatia who reported being in an intimate relationship, sharing a household with the partner (whether married or simply cohabitating), and being mother to a child with a diagnosed developmental disability.

Participants were recruited through educational centres, private rehabilitation practices, as well as formal and informal parent associations and support groups. Initial contact with participants was made through school principals, superintendents, or representatives who had been informed about the study and were asked to send potential participants a link to participate in the surveys (see below). We aimed to engage as many parent’s associations and institutions as possible, specifically those providing care for children with disabilities or their families, across the country. This strategy was designed to involve a broad spectrum of mothers who raise children with disabilities in our research. As most of these institutions are concentrated in Zagreb, the capital city, it’s presumed that the majority of participating mothers reside there. These mothers likely have better access to support services. Furthermore, it’s essential to note another potential bias due to the fact that research only included mothers who responded to the experts’ invitations and accessed the provided links.

The study was approved by the Ethics Committee of the Faculty of Education and Rehabilitation Sciences, University of Zagreb, on 1st of February 2021 (Classification: 602-25/23-01/42; Registry number: 251-74/23-02-7/2).

### 2.2 Survey procedure

Between March and April 2021, potential participants were sent an online link to complete the surveys below via the SurveyMonkey tool. They could complete the questionnaires either on a smartphone or a computer. Participants were prevented from completing the questionnaire more than once with SurveyMonkey default feature. Before being allowed to complete them, potential participants were given basic information about the purpose of the study, objectives and procedures of the research and how the data would be used, as well as assurances that their responses would be analysed anonymously and kept confidential. They were informed that their participation was voluntary and that they could withdraw consent even after completing the questionnaires. Participants were given information about time required to complete the survey (15–20 min). Upon reviewing the fundamental details of the research and carefully reading the instructions, the participants expressed their consent to engage in the study. This affirmation was indicated by their action of clicking on the questionnaire, following which they proceeded to respond to the posed questions. Only after providing this consent, were they allowed to access the questionnaires and submit responses. Since participants completed questionnaires online, information on how many potential participants were reached was not collected and it is not possible to provide details on the response rate.

The link to the surveys was available to participants from March 1, 2021 to April 1, 2021. Given that no new participant responses were observed after approximately 2 weeks, the researchers agreed that the invitation to participate in the research and the link to the questionnaire would be available for another 2 weeks, for a total of 1 month. Participants in our research were not provided with any incentives and participating was on an entirely voluntary basis. However, there was an exclusion criteria: the participants in the research had to be women who have been in a current intimate relationship for at least 6 months.

### 2.3 Surveys

Participants first were asked to provide sociodemographic data about themselves and their child with disabilities, including their age and the child’s age, duration of their relationship with their partner, their education level, their employment status, monthly household income (i.e., joint income contributed by all employed persons in the household), the child’s gender and the child’s disability according to the following six categories defined by the Croatian government (39): intellectual disability; autism spectrum disorder; sensorimotor disability; genetic syndrome or chronic disease; attention deficit hyperactivity disorder, specific learning disability, or sensory integration disorder; or disorder of speech, language, and voice. Parents could check more than one category. In case the participants had more than one child with developmental difficulties, they had the opportunity to answer the questions for each child separately.

Then participants were asked to fill out Croatian versions of the following questionnaires. The Parent Problem Checklist (40), which we translated into Croatian ourselves using a back-translation procedure involving two translators working independently, was used to measure the extent of parental conflict related to childrearing. Given that our study focused on interparental conflicts in general, and that we were interested in exploring number of disagreements and the intensity of the conflicts all parents have, regardless of whether they have a typically developing child or child with disabilities aged 0–18 years, we assessed this instrument as suitable for our research. The checklist includes 16 conflict interactions, including disagreements about discipline or rules, the extent of overt conflict related to childrearing, and mutual undermining of a parent’s relationship with the child. Participants indicated on a scale of 1 (“never”) to 5 (“very often”) how often a given interaction had occurred between them and their partner during the previous 4 weeks. Examples of items include “Disagreements about how to discipline” or “One soft, one hard parent.” The scores on individual items were summed to obtain a total score, with higher score indicating a higher level of conflict between parents. The internal consistency of the scale in the present study was *α* = 0.91, which indicated that items in this instrument measure the same construct and that we could interpret the results indeed as a level of interparental conflicts.

The Dyadic Coping Inventory (41), which we translated into Croatian ourselves using a back-translation procedure involving two translators working independently, was used to measure dyadic coping between the respondent and her partner. The inventory consists of 35 items and includes ratings of the respondent’s own coping, her partner’s coping, and joint coping, referring to when the respondent and her partner faced a stressor jointly (41). The first two types of coping were assessed in terms of supportive coping behaviors (e.g., “My partner listens to me and gives me the opportunity to share what is really bothering me”), delegated coping behaviors (e.g., “When I am too busy, my partner helps me”), and negative coping behaviors (e.g., “When I am stressed, my partner tends to withdraw”). Participants were asked to respond on a scale from 1 (“very rarely”) to 5 (“very often”) about the frequency of these behaviors in general. The scores on individual items were summed to obtain a total score, with greater scores indicating stronger dyadic coping behavior. Subscores were also calculated for each of the three types of coping (own, partner’s and joint). In the present study, internal consistency was *α* = 0.94 for the total scale, while it ranged from 0.64 to 0.91 for the subscales, which is satisfactory. In our study, principal component analysis yielded the three-factor solution for both own and partner’s coping and the predicted one-factor solution for joint/common dyadic coping, which accounted for 55.5, 66.7 and 72.5% of variance, respectively. The potential bias in adapting and validating this instrument in Croatian language could stem from sampling method (non-probabilistic sample that included only women) and characteristics of the sample (mothers of children with disabilities, in monogamous heterosexual relationship). No other potential bias regarding language, culture or other sources of bias was deemed relevant.

The Marriage Quality Index (42) was used to measure relationship satisfaction, as it was already validated in Croatian translation (43). The index includes five items (e.g., “My relationship with my partner makes me happy”), to which participants responded on a scale from 1 (“strongly disagree”) to 7 (“strongly agree”); and one item (“All things considered, how happy are you in your relationship with your partner?”), to which participants responded on a scale from 1 (“very unhappy”) to 10 (“very happy”). Responses to all items were averaged to obtain an overall score. The internal consistency of the scale in the present study was 0.97, which is satisfactory.

### 2.4 Data analysis

Data were analysed using SPSS 28 (IBM, Chicago, IL, USA). Pearson correlation analysis was performed to detect significant associations between overall scores or subscores on the three questionnaires. Hierarchical regression was carried out in order to examine how much sociodemographic variables, interparental conflict, or dyadic coping accounted for the observed variance in relationship satisfaction. Lastly, moderation analysis was conducted to establish if subscales of dyadic coping are moderators in the association between interparental conflicts and relationship satisfaction.

## 3 Results

### 3.1 Descriptive statistics

This study analysed 232 women between 24 and 57 years old (*M* = 39.13, *SD* = 6.19), who reported having been in an intimate relationship with their current partner for an average of 14 years (range, 1–34 years). Just over half of participants (56.9%) had a high school diploma, while 40.9% had a university degree (college degree, master’s degree, or PhD) (Table 1). While 29.3% of mothers were employed full time, 20.7% were working with reduced working hours or part time and majority of them (37.5%) reported having received “parent caregiver” status from the local government or being on maternity leave, which implied that they were not working in order to spend more time with their child with disability. Over half the respondents (62%) reported a monthly household income of 1,000–2000 EUR, while nearly a quarter (22.8%) reported an income below 1,000 EUR.

**Table 1:** Characteristics of the respondents (*N* = 232).

Characteristics of respondents		
	Average	Range
Age, year	39.13	24–57
Duration of intimate relationship, yr	14	1–34
Education	%	
-Elementary school education	2.2	
-High school diploma	56.9	
-College degree	35.3	
-Master’s degree or PhD	5.6	
Employment		
Full time	29.3	
Reduced working hours or part time	20.7	
“Parent caregiver” status or maternity leave	37.5	
Monthly household income		
< 1,000 EUR	22.8	
1,000–2000 EUR	62	
> 2000 EUR	15.1	

Among the respondents’ children with disabilities, their average age was 8 years (*SD* = 4.05; range 1–17 years) and 66.8% were male (Table 2). One third (33.2%) of mothers had a child with multiple disabilities and 20.3% had a child with autism spectrum disorder. Of the other developmental disabilities, the most prevalent were speech, language, and voice disorders (13.4%) and genetic syndromes or chronic diseases (11.2%). Further, 9.9% mothers had a child with attention deficit hyperactivity disorder (ADHD), specific learning difficulties, or sensory integration disorders, while 7.3% had a child with various sensorimotor disabilities. Lastly, only 4.7% mothers in our study had a child with intellectual disability. The majority of children with disabilities in Croatia are integrated into the regular education system, if they do not have multiple or complex disabilities that would prevent them from completing regular curriculum with an individualized approach or curriculum adaptation. Regardless of whether the child is placed in a regular or special educational system, parents of children with disabilities in Croatia lack systematic support in fulfilling their rights and the rights of their children, which is why parents of children with various disabilities often face the same or similar challenges in everyday life and during child’s growing up.

**Table 2:** Characteristics of the respondents’ children with disabilities (*N* = 232).

Characteristics of children with disabilities		
Male	66.8	
Female	33.2	
	Average	Range
Age, year	8	1–17
Type of disability	%	
Multiple types	33.2	
Autism spectrum disorder	20.3	
Speech, language, and voice disorders	13.4	
Genetic syndromes or chronic diseases	11.2	
ADHD, specific learning difficulties, and sensory integration disorders	9.9	
Sensorimotor disabilities	7.3	
Intellectual disabilities	4.7	

ADHD, attention deficit hyperactivity disorder.

The scores of the respondents on the Parent Problem Checklist, Dyadic Coping Inventory and Marriage Quality Index are presented in Table 3. The scores showed a normal distribution based on the fact that the z-scores for skewness and kurtosis fell between −3.29 and 3.29 (44), so parametric statistical tests were used. Since results on the subscales “Supportive dyadic coping by oneself,” “Delegated dyadic coping by oneself,” “Negative dyadic coping by oneself” of the Dyadic Coping Inventory and on the Marriage Quality Index deviated significantly from normal, non-parametric indicators were used (i.e., median and interquartile range). Nonetheless, research has demonstrated that the Pearson correlation coefficient remains reliable even when applied to skewed distributions, indicating its validity in such cases (45). The mean score on interparental conflict was 37.39 (*SD* = 12.13) indicating moderate frequency and intensity of disagreements with the partner, such as when discussing parenting decisions. The total mean score for dyadic coping was 123.72 (*SD* = 21.55), which falls within the normal range of 111–145 (46). When it comes to mothers’ perception of their own coping with stress (“Supportive dyadic coping by oneself,” “Delegated dyadic coping by oneself”), they perceive themselves as highly supportive toward their partner when he deals with stressful situations (*C* = 20, *Q_1_* = 18, *Q_3_* = 22 for supportive and *C* = 8, *Q_1_* = 6, *Q_3_* = 8 for delegated coping). On the other hand, mothers’ perceptions of their own negative dyadic coping are lower (*C* = 8, *Q_1_* = 6, *Q_3_* = 10) which indicates they rarely perceive themselves as being unsupportive, e.g., displaying hostile or superficial behavior toward their partner when he is going through stressful situations. Mean scores on the subscales “Supportive dyadic coping of the partner” and “Delegated dyadic coping of the partner” were, respectively, 16.72 (*SD* = 6.26) and 6.59 (*SD* = 2.33), indicating that most mothers in our study generally perceived their partners to be supportive in times of stress. Mean score on the subscale “Common dyadic coping” was 16.14 (*SD* = 4.90), implying that mothers’ perception of how she and her partner cope together with shared stressful situations is successful and symmetrical. Median score on the Marriage Quality Index was 6.50 (*Q_1_* = 5.33, *Q_3_* = 7.33), representing that respondents were very satisfied with their intimate relationship.

**Table 3:** Questionnaire scores for 232 mothers raising children with disabilities.

	Min	Max	*M*	SD		S–W	SKEW	Z_s_	KURT	Z_k_
Parenting problem checklist										
Interparental conflict	6	73	37.39	12.13		0.98**	0.47	2.91	−0.02	−0.05
Dyadic coping inventory										
Supportive dyadic coping of the partner	5	25	16.72	5.26		0.96**	−0.49	−3.04	−0.61	−1.92
Delegated dyadic coping of the partner	2	10	6.59	2.33		0.93**	−0.51	−3.20	−0.57	−1.79
Negative dyadic coping of the partner	4	20	9.51	3.74		0.96**	−0.50	−3.11	−0.39	−1.22
Common dyadic coping	5	25	16.14	4.90		0.97**	−0.32	−2.00	−0.57	−1.78
Total score	59	172	123.72	21.55		0.99*	−0.30	−1.90	−0.23	−0.72
	Min	Max	*C*	Q_1_	Q_3_	S–W	SKEW	Z_s_	KURT	Z_k_
Supportive dyadic coping by oneself	7	25	20	18	22	0.96**	−0.69	−4.31	1.50	4.73
Delegated dyadic coping by oneself	2	10	8	6	8	0.93**	−0.67	−4.16	0.85	2.68
Negative dyadic coping by oneself	4	17	8	6	10	0.95**	−0.54	−3.39	0.10	0.33
Marriage quality index										
Relationship satisfaction	1	8	6.50	5.33	7.33	0.84**	−1.27	7.91	0.78	2.46

*M*, mean; SD, standard deviation; S–W, Shapiro–Wilk test; SKEW, Skewness; Z_s_, z-score of skewness; KURT, kurtosis; Z_k_, z-score of kurtosis; *p < 0.05; **p < 0.01.

### 3.2 Correlations

Pearson correlation analysis revealed several associations between scores on the three questionnaires (Table 4). Interparental conflict, as expected, correlated negatively with “Supportive dyadic coping by oneself” (*r* = −0.22, *p* < 0.01), “Supportive dyadic coping of the partner” (*r* = −0.54, *p* < 0.01), “Delegated coping of the partner” (*r* = −0.44, *p* < 0.01) and “Common dyadic coping” (*r* = −0.53, *p* < 0.01). Results showed interparental conflict did not correlate with “Delegated dyadic coping by oneself.” These results suggest that respondents who had more frequent conflicts with their partner about parenting also perceived their communication with the partner to be worse and their ability to cope with stress as a couple to be weaker. Parents experiencing frequent disagreements in raising children with disabilities may encounter heightened stress levels, limiting their capacity to support their partner during stressful events. Moreover, when one parent perceives the other’s parenting views as insignificant, they might struggle to empathize during their partner’s stress. In conflictual situations, partners might overlook each other’s attempts to help them cope with stress, reducing the likelihood of future supportive dyadic coping. Additionally, couples not accustomed to employ supportive dyadic coping, or employing negative coping, may face more overall negative interactions in a relationship. This can manifest in arguments in the presence of children, unresolved conflicts, or divergent disciplinary approaches. Finally, interparental conflict correlated negatively with relationship satisfaction (*r* = −0.60, *p* < 0.01). In other words, mothers who reported more frequent conflicts with their partner over their child disability perceived their dyadic stress management as worse and their intimate relationship with their partner as less satisfying.

**Table 4:** Pearson correlations between overall scores or subscores on the three questionnaires.

	Interparental conflict	SDC by oneself	DDC by oneself	NDC by oneself	SDC of the partner	DDC of the partner	NDC of the partner	Common dyadic coping	Relationship satisfaction
Interparental conflict	–								
SDC by oneself	−0.22**	–							
DDC by oneself	−0.11	0.54**	–						
NDC by oneself	0.42**	−0.42**	−0.22**	–					
SDC of the partner	−0.54**	0.42**	0.19**	0.39**	–				
DDC of the partner	−0.44**	0.32**	0.27**	0.30**	0.73**	–			
NDC of the partner	0.66**	−0.25**	−0.07	0.47**	−0.64**	−0.46**	–		
Common dyadic coping	−0.53**	0.44**	0.31**	0.33**	0.76**	0.60**	0.55**	–	
Relationship satisfaction	−0.60**	0.32**	0.17**	0.42**	0.78**	0.65**	0.68**	0.72**	–

SDC, Supportive Dyadic Coping, DDC, Delegated Dyadic Coping, NDC, Negative Dyadic Coping; **p < 0.01.

Conversely, relationship satisfaction correlated positively with common dyadic coping (*r* = 0.72, *p* < 0.01) and all subscales of coping by oneself and coping of the partner. The highest correlation was found between relationship satisfaction and “Supportive dyadic coping of the partner” (*r* = 0.78, *p* < 0.01) and the lowest between relationship satisfaction and “Delegated dyadic coping by oneself” (*r* = 0.17, *p* < 0.01). These results suggest that mothers of children with disabilities who are more satisfied with their relationship with their partner also are more likely to perceive their partner as caring, helpful, and supportive in stressful times. Also, how couples cope with stress together as a team appears to be crucial for maintaining a happy, fulfilling intimate relationship.

### 3.3 Hierarchical multiple regression analysis

Hierarchical multiple regression analysis was conducted to identify which variables contributed to relationship satisfaction of mothers of children with disabilities (Table 5). In the first step, the predictors were sociodemographic variables (respondent age, duration of intimate relationship); in the second step, the predictor was interparental conflict; and in the third step, the predictors were subscales of Dyadic Coping Inventory. The entire set of predictors explained 71.7% of the observed variance in relationship satisfaction, which was statistically significant [*F* (10, 221) = 55.988, *p* < 0.01]. Each of the three steps explained a significant proportion of the variance: first step 3.3% (*p* < 0.05), second step 37.2% (*p* < 0.01) and the third step 71.7% (*p* < 0.01). In the first step, both predictors, respondent age (*β* = −0.24, *p* < 0.01) and duration of intimate relationship (*β* = 0.23, *p* < 0.05) were significant. These results indicate that older mothers in our study were less satisfied with their relationship, as often happens when partners enter middle age and use resources to cope with the challenges of everyday life related to careers and childcare (47). On the other hand, those mothers who were in their relationship for a longer period were also more satisfied with the relationship, which means that partners over time invest in their relationship, grow closer to each other, and develop closeness (48) which is related to their levels of satisfaction and commitment (49).

**Table 5:** Hierarchical multiple regression to identify contributions of study variables to relationship satisfaction.

Predictors	*β*
Step 1 – Sociodemographic variables	
Respondent age	−0.24** (−0.14) [0.00]
Duration of intimate relationship	0.23* (0.16*) [0.03]
Step 2 – Interparental conflict	
Interparental conflict	(−0.59**) [−0.10*]
Rc^2^	0.37**
ΔR^2^	0.34**
Step 3 – Dyadic coping subscales	
Supportive dyadic coping by oneself	[−0.06]
Delegated dyadic coping by oneself	[−0.01]
Negative dyadic coping by oneself	[−0.08]
Supportive dyadic coping of the partner	[0.31**]
Delegated dyadic coping of the partner	[0.16**]
Negative dyadic coping of the partner	[−0.19**]
Common dyadic coping	[0.24**]
Rc^2^	0.72**
ΔR^2^	0.35**

*β*, standardized regression coefficient; (), values of standardized regression coefficient in Step 2; [], values of standardized regression coefficient in Step 3; Rc^2^, adjusted multiple determination coefficient; ΔR^2^, change in proportion of explained variance; *p < 0.05; **p < 0.01.

In the second step, interparental conflicts (*β* = −0.59, *p* < 0.01) were introduced, which resulted in respondent age being no longer statistically significant predictor of relationship satisfaction, while duration of intimate relationship was still significant predictor (*β* = 0.16, *p* < 0.05). Higher satisfaction with the intimate relationship was associated with less frequent conflict between parents. This means that interactions between partners, such as interparental conflicts, explain some of the variance of age in predicting relationship satisfaction. Also, it means that for mothers of children with disabilities, when it comes to their satisfaction with intimate relationship, it is more important how mothers cooperate and communicate with their partner while raising children together.

Finally, in the third step, subscales of dyadic coping were introduced, which resulted in duration of intimate relationship becoming insignificant as a predictor. Interestingly, none of the subscales of dyadic coping by oneself (supportive, delegated, or negative) predicted relationship satisfaction of mothers of children with disabilities in our study. However, all of the subscales of dyadic coping of the partner, as well as common dyadic coping, were predictors of relationship satisfaction. The strongest predictors were “Supportive dyadic coping of the partner” (*β* = 0.31, *p* < 0.01) and “Common dyadic coping” (*β* = 0.24, *p* < 0.01). “Delegated dyadic coping of the partner” was somewhat weaker, but still statistically significant predictor of the relationship satisfaction (*β* = 0.16, *p* < 0.01). As expected, “Negative dyadic coping of the partner” was negative predictor of the relationship satisfaction (*β* = −0.19, *p* < 0.01). These results indicate that mothers’ perceptions of how they help their partner cope with stress do not predict how satisfied are they in a relationship; instead, relationship satisfaction depends on their perceptions of how their partner helps them and how they cope with stress as a team. Also, hostile, and ambivalent interactions with the partner, e.g., partner withdrawing in stressful situations, partner providing support, but doing it unwillingly etc. have a deleterious effect and predict lower levels of relationship satisfaction in mothers. Two studies (26, 50) found that the perceived marital quality of women from the general population depended on both their own and their partner’s dyadic coping, whereas the same parameter among men depended only on their own dyadic coping. These two previous studies and the present work strengthen the idea that mothers raising a child with disabilities place a premium on the quality of support they receive from their partners.

### 3.4 Moderation analysis

In order to examine whether the correlation between interparental conflict and relationship satisfaction in mothers of children with disabilities depends on dyadic coping, regression analyses were conducted on centered variables of interparental conflict and subscales of dyadic coping (including the interaction of these variables), where the confidence intervals were determined by the bootstrap method on 1,000 samples. As can be seen from Table 6, none of the dyadic coping of oneself subscales moderated the correlation between interparental conflict and relationship satisfaction of mothers in our study, while only “Supportive dyadic coping of the partner” and “Negative dyadic coping of the partner” moderated the correlation between interparental conflict and relationship satisfaction of mothers in our study (Table 7).

**Table 6:** Moderation analysis results for DC by oneself.

Moderation estimates			95% Confidence interval			
	Estimate	SE	Lower	Upper	*Z*	*p*
Interparental conflict	−0.07846	0.00904	−0.0956	−0.0592	−8.67	< 0.001
Supportive dyadic coping by oneself	0.10727	0.03749	0.0374	0.1828	2.86	0.004
Interparental conflict ✻ Supportive dyadic coping by oneself	−0.00520	0.00300	−0.0108	9.10e-4	−1.73	0.083
Interparental conflict	−0.0800	0.00877	−0.0980	−0.06389	−9.12	< 0.001
Delegated dyadic coping by oneself	0.1010	0.06071	−0.0205	0.21832	1.66	0.096
Interparental conflict ✻ Delegated dyadic coping by oneself	−0.0132	0.00676	−0.0250	0.00114	−1.96	0.051
Interparental conflict	−0.06937	0.01025	−0.08857	−0.04849	−6.767	< 0.001
Negative dyadic coping by oneself	−0.12236	0.04028	−0.20338	−0.04868	−3.037	0.002
Interparental conflict ✻ Negative dyadic coping by oneself	−0.00166	0.00261	−0.00720	0.00380	−0.636	0.525

DC, dyadic coping.

**Table 7:** Moderation analysis results for DC of the partner and common DC.

Moderation estimates			95% Confidence interval			
	Estimate	SE	Lower	Upper	*Z*	*p*
Interparental conflict	−0.02859	0.00839	−0.0443	−0.01129	−3.41	< 0.001
Supportive dyadic coping of the partner	0.20312	0.01914	0.1644	0.24121	10.61	< 0.001
Interparental conflict ✻ Supportive dyadic coping of the partner	0.00293	0.00130	3.14e-5	0.00522	2.26	0.024
Interparental conflict	−0.05373	0.00831	−0.07028	−0.03819	−6.465	< 0.001
Delegated dyadic coping of the partner	0.34870	0.04673	0.25413	0.43906	7.462	< 0.001
Interparental conflict ✻ Delegated dyadic coping of the partner	0.00110	0.00338	−0.00564	0.00779	0.326	0.745
Interparental conflict	−0.03264	0.01209	−0.05523	−0.00725	−2.70	0.007
Negative dyadic coping of the partner	−0.21269	0.03506	−0.28185	−0.14801	−6.07	< 0.001
Interparental conflict ✻ Negative dyadic coping of the partner	−0.00446	0.00166	−0.00733	−7.87e−4	−2.68	0.007
Interparental conflict	−0.03739	0.00987	−0.0566	−0.01770	−3.79	< 0.001
Common dyadic coping	0.19055	0.02454	0.1474	0.24247	7.77	< 0.001
Interparental conflict ✻ Common dyadic coping of the partner	0.00234	0.00145	−6.69e−4	0.00509	1.61	0.108

DC, dyadic coping.

**Figure 1:**
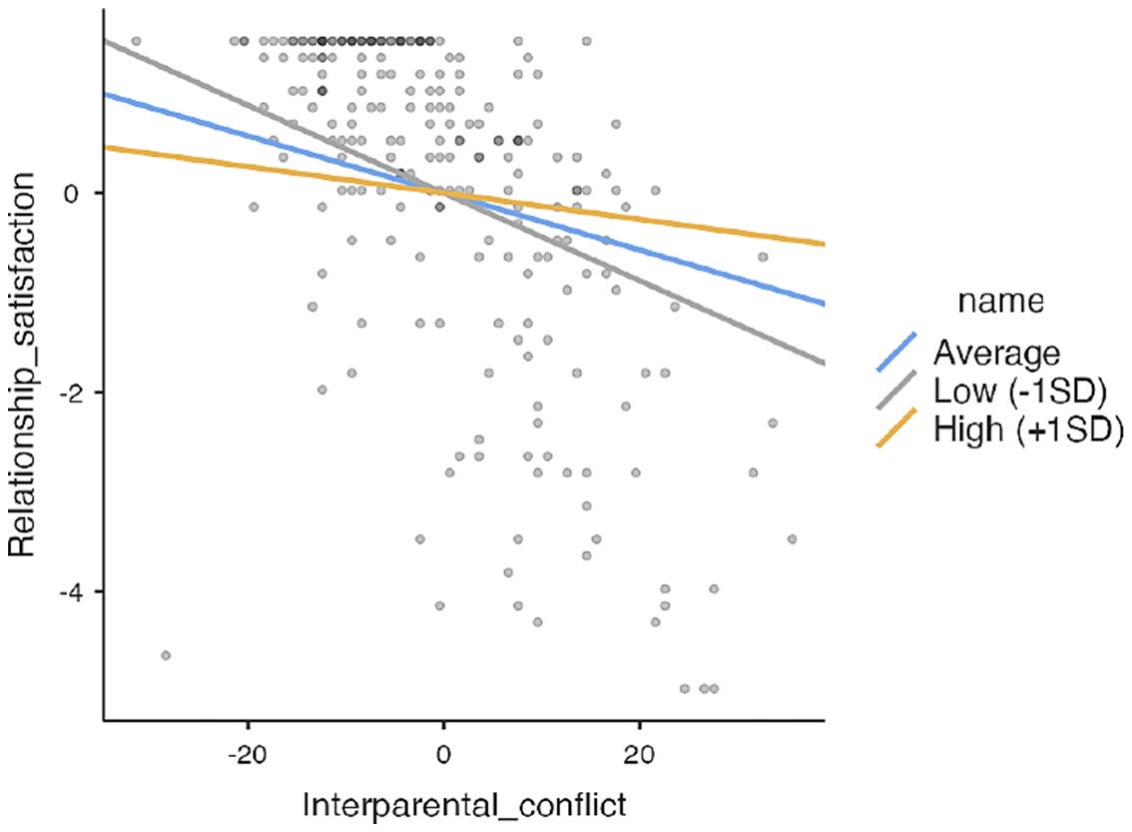
Moderating role of supportive dyadic coping of the partner.

When it comes to “Supportive dyadic coping of the partner” (b_interaction_ = 0.003, 95%CI [0.0001, 0.005]), interparental conflict was a significant predictor of the lower relationship satisfaction for mothers of children with disabilities, but only when moderator, in this case supportive dyadic coping, was average or lower (–1SD), as can be seen from Table 7. In those mothers who perceive their partner as highly supportive, i.e., showing empathy, helping analyze the stressful situation, giving opportunity to communicate etc., interparental conflicts were not predicting decrease in relationship satisfaction. In other words, the effect of interparental conflicts was entirely mitigated by having a supportive partner with whom mothers could share stress (Figure 1).

**Figure 2:**
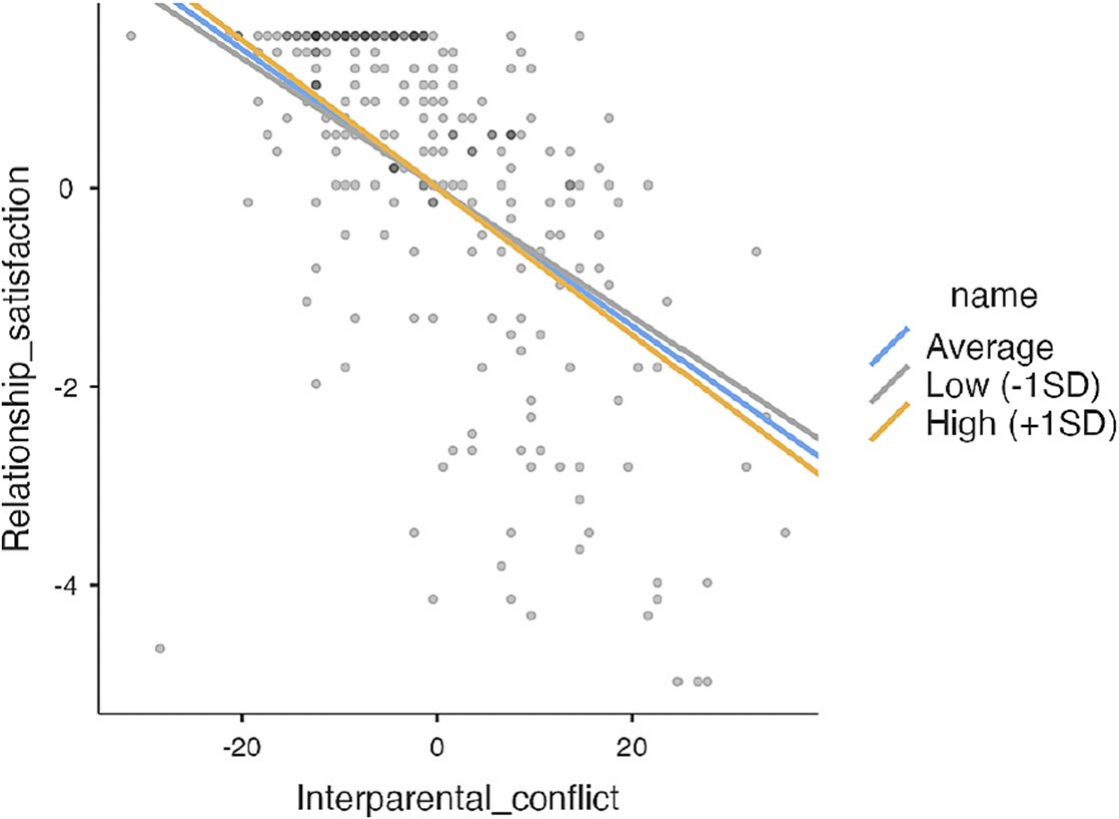
Moderating role of negative dyadic coping of the partner.

“Negative dyadic coping of the partner” (b_interaction_ = −0.004, 95%CI [−0.007, −0.0007]), interparental conflict negatively predicted relationship satisfaction for mothers of children with disabilities, but only when moderator, in this case negative dyadic coping, was average or high (+1SD). In those mothers who perceive more negative interactions with their partner during stressful situations (i.e., blaming, withdrawing, not taking stress seriously etc.), interparental conflicts will decrease relationship satisfaction. However, if partner does not cope with stress negatively, interparental conflicts will not have a negative effect on relationship satisfaction (Figure 2).

Thus, in the case of mothers of children with developmental disabilities, the satisfaction with the partner relationship contributes the most to her satisfaction with the support her partner provides her in stressful situations. When these mothers feel adequately supported by their partner, the negative impact of stress caused by conflicts related to their children does not spill over into their relationship (Table 8).

**Table 8:** Simple slopes analysis.

Simple slopes estimates – supportive dyadic coping of the partner^1^			95% Confidence interval			
	Estimate	SE	Lower	Upper	*Z*	*p*
Average	−0.0286	0.00833	−0.0438	−0.01125	−3.43	< 0.001
Low (–1SD)	−0.0440	0.01216	−0.0655	−0.01717	−3.62	< 0.001
High (+1SD)	−0.0132	0.00915	−0.0332	0.00309	−1.44	0.149
Simple Slopes Estimates – Negative dyadic coping of the partner^2^			95% Confidence Interval			
	Estimate	SE	Lower	Upper	*Z*	*p*
Average	−0.0326	0.0122	−0.0557	−0.00789	−2.68	0.007
Low (–1SD)	−0.0160	0.0129	−0.0409	0.00878	−1.24	0.216
High (+1SD)	−0.0493	0.0145	−0.0754	−0.01850	−3.40	< 0.001

^1^Shows the effect of the predictor (Interparental conflict) on the dependent variable (Relationship satisfaction) at different levels of the moderator (Supportive dyadic coping of the partner). ^2^Shows the effect of the predictor (Interparental conflict) on the dependent variable (Relationship satisfaction) at different levels of the moderator (Negative dyadic coping of the partner).

## 4 Discussion

Parents of children with disabilities often experience higher levels of parenting stress than parents of children without disabilities (8, 10), which can negatively impact parenting effectiveness (16). Mothers of children with disabilities often function as primary caregivers, so they also face additional, specific sources of stress related to caring for a child with disabilities (19). This study examined the associations of interparental conflict, dyadic coping and certain sociodemographic factors with relationship satisfaction among mothers raising children with developmental disabilities. Our first hypothesis (H1) that mothers’ age and duration of intimate relationship, interparental conflicts and dyadic coping will predict relationship satisfaction of mothers of children with disabilities was partially confirmed. Our expectation was that relationship duration would negatively predict relationship satisfaction (H1.1); however, our results show that is positively predicts it. Also, results show that relationship satisfaction decreased with respondents’ age, but longer relationship duration predicted higher levels of relationship satisfaction, which is consistent with a study (i.e., systematic review and meta-analysis) conducted in 2021 (51), showing that relationship satisfaction in people generally decreases between ages 20 and 40 and then increases up to age of 65; also, satisfaction decreases in the first 10 years and increases up to 20 years of relationship.

Our results could be better understood when we look at surveys of the general population (e.g., 52–54) that show how with increasing age, the satisfaction of women (especially after the birth of children) decreases more than that of men. On the other hand, the more partners invest in relationship, the more likely are they to stay committed, which is consistent with Rusbult’s investment model (55). It is assumed that investment will also increase over time. Our results could indicate that mothers of children with disabilities do not differ from the general population in this respect.

Furthermore, we hypothesized that mothers’ age, duration of intimate relationship and interparental conflict would negatively predict relationship satisfaction, with interparental conflict being the strongest predictor. After we included interparental conflict in second step in hierarchical multiple regression, the effect of mothers’ age becomes insignificant and interparental conflict proved to be a stronger predictor than duration of intimate relationship. This could mean that, over time, interactions between partners become more important for overcoming specific child-rearing challenges and for mothers’ satisfaction with relationship. However, since even after accounting for interparental conflict, the duration of the relationship was still a predictor of greater relationship satisfaction, it appears that mothers who have been in the relationship longer are more likely to invest in it and are also more satisfied with it.

Additionally, mothers in our study who perceived more frequent conflict with their partner regarding children also perceived poorer dyadic coping and were less satisfied with their intimate relationship. Our results are consistent with the idea that stresses around parenting can “spill over” into the intimate relationship between the parents (e.g., 27, 21), reducing relationship satisfaction over time (e.g., 31). This spillover likely reflects multiple processes: first, the parents spend less time together as partners, limiting opportunities for positive couple behaviors such as affection or intimacy (56); and second, the capacity for self-regulation decreases, facilitating the expression of negative individual traits such as aggressiveness and increasing the frequency of conflict and negative couple behaviors. In other words, when partners feel stressed, they engage in fewer positive and more negative interactions with each other, which affects relationship quality, satisfaction, and stability over time. Indeed, lower levels of dyadic coping have been associated with lower relationship stability (57).

Moreover, we hypothesized that subscales of dyadic coping (supportive and delegated coping of oneself and of the partner) will positively predict relationship satisfaction (H1.2), while negative dyadic coping will negatively predict it (H.1.3). This hypothesis was partially confirmed since we found that relationship satisfaction was positively predicted only by supportive and delegated dyadic coping of the partner, but not of oneself; also, it was negatively predicted only by negative dyadic coping of the partner, but not of oneself. Support received from the partner is also deemed important for the perceived marital quality of women in the general population (26). In fact, one study (50) concluded that dyadic coping is more important to women than men for relationship satisfaction. These findings may reflect cultural expectations that mothers and fathers have internalized. In many Western countries, traditional gender roles are still present, and mothers continually take the most care of the children (31). Croatia is not an exception in this regard, since it is traditionally Catholic country, confirmed by the data of 2021 census; out of a little bit over 3.8 million inhabitant living in Croatia, 78.9% identify themselves as Catholics (58). One longitudinal study (59) conducted in Croatia looked at the roles of women and men in the period of 1999–2016 and found that women achieved a positive shift in the level of equality outside the home (i.e., in profession and at workplace), but deteriorated at home, especially in connection with raising children and caring for elderly family members, as well as fulfilling household duties. While men are participating more in the housework, they participate somewhat less in activities related to children. This could mean that women still have to devote most of their free time to the family.

The cultural context in Croatia is somewhat in line with the results of our research, in which out of 232 mothers, 88 were currently unemployed or not actively participating in the workforce (e.g., due to maternity leave or “parent caregiver” status) and 116 were working, of which only 68 full-time and 48 part-time. As our results showed, mothers that were employed full-time had significantly more interparental conflict with their partners, as opposed to mothers that were on maternity leave or had “parent caregiver” status. Considering the extensive involvement of mothers in household and childcare responsibilities, it’s reasonable to anticipate that the support from their partners holds significant importance, whether it’s practical assistance or emotional support. For this reason, relationship satisfaction of mothers in our study was predicted only by partner’s supportive, delegated and negative dyadic coping, while supportive, delegated and negative coping of mothers themselves was not significant in predicting changes in relationship satisfaction.

In concordance with our second hypothesis (H2), dyadic coping indeed moderates the association between interparental conflict and relationship satisfaction. More precisely, supportive dyadic coping of the partner mitigated the association between above mentioned variables (H2.1), while negative dyadic coping of the partner intensifies it (H2.2). Again, common dyadic coping as well as supportive, delegated and negative coping of oneself did not moderate the association between interparental conflict and relationship satisfaction. When mothers rate dyadic coping of the partner as high, the unfavorable effect of interparental conflict do not spill over into relationship with their partner. Conversely, when mothers do not perceive support from their partners (or perceive negative dyadic coping of the partner), interparental conflict results in lower relationship satisfaction.

On the one hand, when mothers are aware of their partner’s support in stressful situations, they may rely more on the father’s parenting skills, which can reduce interparental conflict (31). On the other hand, if mothers perceive their partners’ practical and emotional support, in situations of interparental conflict, the effect of stress spillover into the intimate relationship will not occur and mothers will continue to be satisfied in the relationship. It could be that mothers’ perception of understanding and support from their partner will foster a sense that their partner takes them seriously and cares about them, which provides a sense of belonging and connectedness with partner, strengthens the partner subsystem, and protects it from the external sources of stress.

However, when mothers have the impression that their partner often does not support them while experiencing stressful situations, i.e., if dyadic coping of the partner is negative, then the unfavorable influence of interparental conflict spills over time into the partner relationship. On the contrary, when mothers do not perceive negative dyadic coping of their partners, interparental conflict will not influence relationship satisfaction.

When it comes to long-term implications for the well-being of the whole family, it can be assumed that interparental conflict, dyadic coping and relationship satisfaction play a big role in shaping family dynamics. Benefits for mothers in terms of increased relationship satisfaction, supportive dyadic coping presented by the partner, and less frequent interparental conflict could lead to more understanding between partners. Consequently, this would mean, e.g., equal sharing of household and child-related tasks between partners, as well as more free time for mothers to engage in other activities, such as professional development. Fathers of children with disabilities are often more focused on work, i.e., career and providing for family financially (60), which could lead to them feeling excluded and less competent in parenting role (45). The more involved fathers are in child upbringing, the more opportunities they have for an active and quality parenting and intimate relationship. Greater satisfaction in intimate relationship can influence family dynamics by promoting greater cohesion between family members and focusing on a common goal, which are important protective factors that foster family’s resilience to stress (46).

When it comes to children, studies show they have fewer psychological problems and greater social competence if they grow up in families that support each other and cope with problems together. They also have a sense of emotional security and learn prosocial behaviors in stressful situations as well as strategies of joint problem solving (61). Dyadic coping between partners is related to children’s prosocial behavior (62); positive interactions between parents contribute to child’s well-being (63). On the other hand, poorer dyadic coping leads to internalizing and externalizing symptoms in children (61). Although these findings stem from research in general population, we can assume it is a good place to start to from guidelines for families of children with disabilities as well.

In Croatia, families of children with disabilities are faced with complex challenges and are in need of systematic and continuous external support. Longitudinal study (31) that looked at interrelationship between dyadic coping and interparental conflict in couples comprised 150 couples divided into three groups; one group participated in a couple-oriented program (CCET), the second in a parenting training program (Triple P) and the third was control group. Mothers in first two groups reported greater pre-post increase in dyadic coping when compared to mothers in control group. In addition, mothers that participated in couple-oriented program showed the greatest pre-post decrease in interparental conflict compared to mothers in the other two groups. Results of this study highlight the importance of evidence-based couple-oriented programs, which unfortunately do not exist in Croatia. The majority of formal support for families of children with disabilities is offered by institutions and non-government organizations in the form of parenting programs designed to teach parenting skills; to a lesser extent they provide parents with individual psychological support. Again, mostly mothers take part in such programs.

Parents of children with disabilities are often unaware of the importance of the relationship quality because a child with disability is often their primary focus. In Croatia, parents of adults with disabilities reported that they lack professional support for maintaining partner relationships (64). The most important support for parents of children with disabilities is one obtained by family members, especially from the partner/spouse (65). It is followed by informal support from other family members, friends and relatives, and only then comes the formal support which is insufficient and with which parents are dissatisfied. Moreover, support from other parents who raise children with disabilities is also of great importance for parents, which is in line with other studies (66).

The aim of external support network is to help parents deal with children’s challenging behaviors, while also promoting children’s learning and developmental progress, in order to reduce stress for parents and thus decrease frequency and intensity of interparental conflict. To achieve this, it would be important to include parents in support groups and/or programs that directly affect the quality of intimate relationship, such as communication between partners, personal and mutual coping with stress and coping with conflict. Through training, education, counseling, partner psychotherapy and support groups, partners can increase positive interactions, reduce conflicts and develop greater satisfaction in their relationship. Since practical experience shows that it is challenging to include both parents in those kinds of programs, implementing support programs should be followed by providing childcare, so chances of involving both parents are increased.

## 5 Limitations of the study

Our study has some limitations that could potentially affect the generalizability of the results. First, our study only examined mothers, their perception of interparental conflict and dyadic coping and relationship satisfaction. We did not examine the relationship dyad from the fathers’ perspective. Since fathers are an understudied population group in both intimate relationships and parenting (this is even more true for parents of children with disabilities), future studies should fill this gap. In some families, fathers have almost as much responsibility for parenting as the mother and may even be the child’s primary caregiver. In addition, parents often disagree on child-related issues and perceive stress and conflict with their partners differently. Therefore, studies should use dyadic designs that include the perspective of both partners simultaneously. In this way, relationship phenomena, such as relationship satisfaction and common/joint coping, would be addressed at the interpersonal level – as it should be – and not at the intrapersonal level. There is evidence that the response of partners to a parent’s “relationship-oriented coping” in raising children with disabilities can strongly influence the parent’s psychological distress (38). For example, if one parent makes an effort to compromise and empathize in stressful caregiving situations, but the partner does not reciprocate, the first parent may suffer more stress.

Second, we did not examine perceived parenting stress in our study. Future studies should include such measures and differentiate between acute and chronic stress. Furthermore, the DCI was used non-specifically and was not specifically associated with child-related stress. Therefore, conclusions about mothers’ dyadic coping (and perceived coping of their partners) in relation to child-related stress should be interpreted with caution. We did not measure the presence of other stressors that might also cluster with child-related stressors. Future studies should therefore be strengthened with more detailed stress measures.

Third, our study did not examine the potential moderator effect of the diverse diagnosis of the child, the age of the child with disabilities, or perceived support from other family members. This should be considered when planning further research. For example, we would expect the two variables mentioned to have a potential impact on relationship satisfaction for both mothers and fathers. In addition to examining the potential moderators, studies should also perform assessments longitudinally rather than only once, as in the present work, as relationship satisfaction may fluctuate considerably overtime, especially in higher stress situations such as parenting children with disabilities (23).

With regard to generalizability of the study, we should also mention that the mothers in our study were generally very satisfied with their relationship and also rated their dyadic coping as good (or average). Therefore, we cannot generalize the results to those mothers/parents who are less satisfied or do not cope as well with stress. Furthermore, the mothers in our sample do not have particularly high interparental conflicts with their partners – the conflicts are moderately frequent, so this also limits the conclusions on our specific population. Quite a number of mothers are not actively employed (e.g., on maternity leave), and quite a number of them also work part-time, which also means that they can provide most of the childcare, but also limits the generalizability of results in our study.

## Data availability statement

The raw data supporting the conclusions of this article will be made available by the authors, without undue reservation.

## Ethics statement

The study was approved by the Ethics Committee of the Faculty of Education and Rehabilitation Sciences, University of Zagreb.

## Author contributions

MVP: Conceptualization, Data curation, Formal analysis, Investigation, Methodology, Project administration, Writing – original draft, Writing – review & editing. AWJ: Conceptualization, Funding acquisition, Investigation, Validation, Writing – original draft, Writing – review & editing. AL: Conceptualization, Data curation, Investigation, Project administration, Validation, Writing – original draft.
